# Supplementary material for: ADAMTS7 promotes smooth muscle foam cell expansion in atherosclerosis
Source: J Clin Invest. 2026 Jan 29;136(6):e187451. doi: 10.1172/JCI187451 (PMC12987627; doi:10.1172/JCI187451)
Supplement: Supplemental data [file jci-136-187451-s208.pdf]

SUPPLEMENTAL FIGURE 1

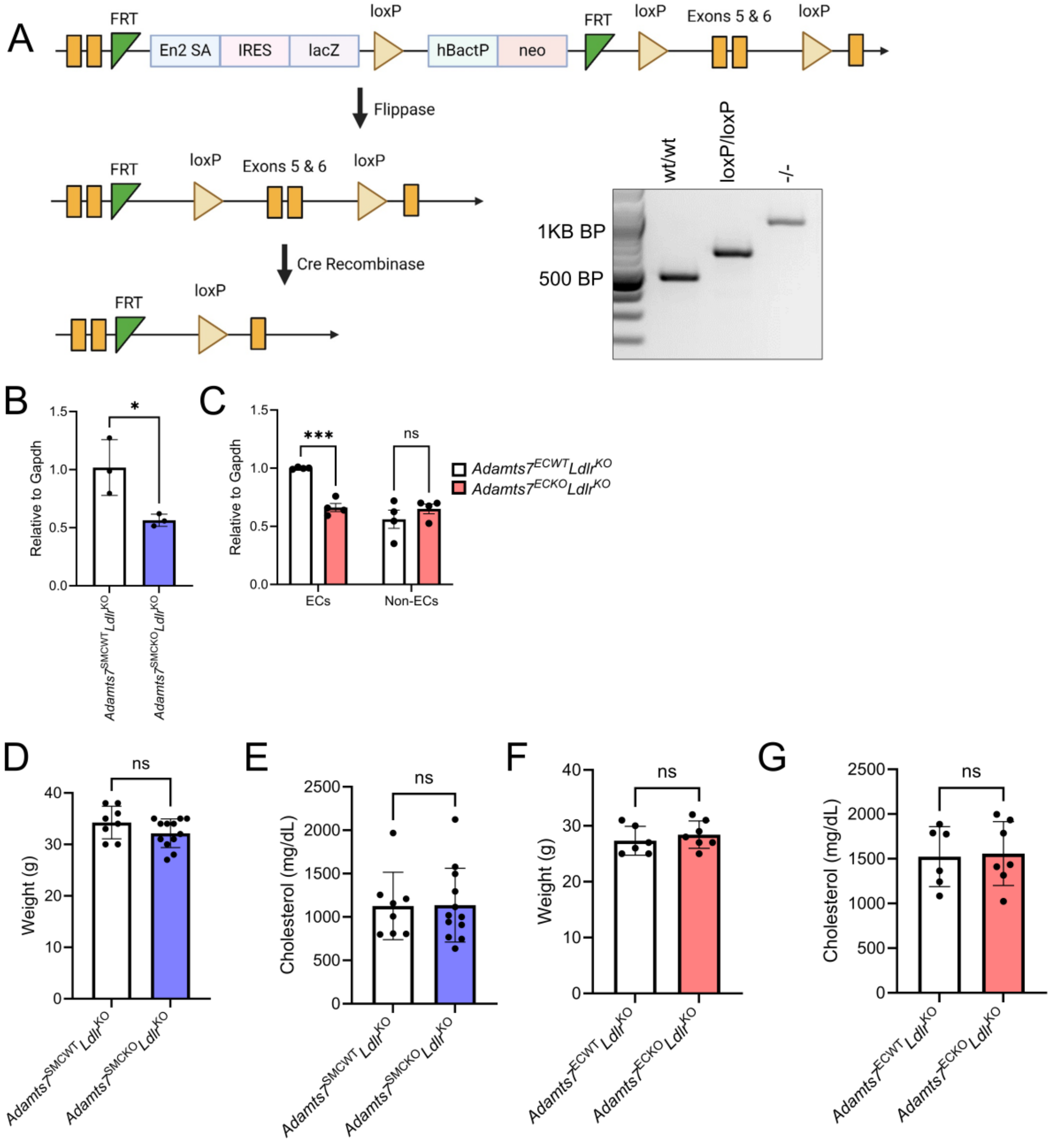

Supplemental Figure 1. Design of conditional knockout mice and weights and cholesterol after WTD. (A) Schematic overview of the knockout-first allele. Knockout mice were crossed with the flp recombinase deleter mice, generating mice with floxed *Adamts7* exons 5 and 6. Subsequently, breeding the conditional knockout mice with a Cre recombinase produces tissue-specific *Adamts7* knockout. Genotyping of wild-type, conditional knockout, and whole-body knockout mice. (B) Primary SMCs were explanted from *Adamts7*^{SMCWT} *Ldlr*^{KO} and *Adamts7*^{SMCKO} *Ldlr*^{KO} mice following tamoxifen induction and analyzed for *Adamts7* expression (*n* = 3 mice). (C) Verification of *Adamts7* knockout in CD31 bead-purified mouse aortic endothelial (CD31^{+}) and non-endothelial (CD31^{-}) cells (*n* = 4). (D) Mice's weight (grams) and (E) plasma total cholesterol after 16 weeks of WTD feeding for *Adamts7*^{SMCWT} *Ldlr*^{KO} and *Adamts7*^{SMCKO} *Ldlr*^{KO} mice. *n* = 8 - 12 male mice. (F) Mice's weight (grams) and (G) plasma total cholesterol after 16 weeks of WTD feeding for *Adamts7*^{ECWT} *Ldlr*^{KO} and *Adamts7*^{ECKO} *Ldlr*^{KO} mice. *n* = 6 - 7 female mice. Statistics were analyzed using a 2-tailed Student's t-test. *$P < 0.05$; ***$P < 0.001$

SUPPLEMENTAL FIGURE 2

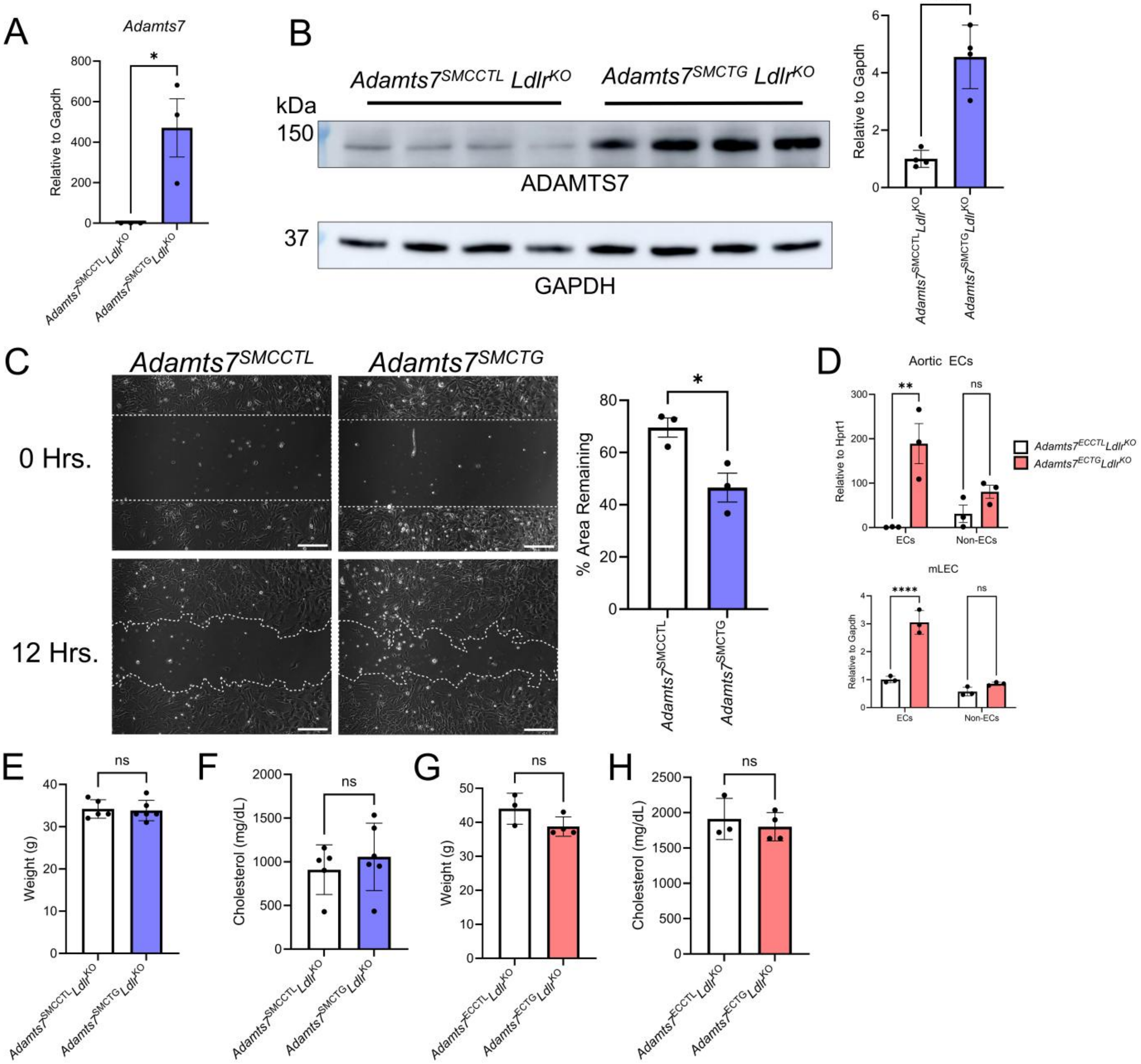

Supplemental Figure 2. Verification and validation of transgenic mice. (A) Verification of *Adamts7* overexpression in Adamts7^{SMCTG} Ldlr^{KO} mice by qPCR *n* = 3 male mice. (B) Validation of ADAMTS7 protein overexpression *n* = 4 male mice. (C) *Adamts7*^{SMCTG} mice have enhanced migration. *n* = 3 male mice. Scale bar = 200 µm (D) Confirmation of *Adamts7* overexpression in bead-purified aortic endothelial (CD31^{+}) and non-endothelial (CD31^{-}) cells from EC-Adamts7 transgenic mice (*n* = 3 mice). Verification of *Adamts7* transgenic expression in isolated mouse lung ECs (CD31^{+}) and non-ECs (CD31^{-}) *n* = 3 mice. (E) Mice's weight (grams) and (F) plasma total cholesterol after 16 weeks of WTD feeding for *Adamts7*^{SMCCTL} *Ldlr*^{KO} and *Adamts7*^{SMCTG} *Ldlr*^{KO} mice. *n* = 5 - 7 male mice. (G) Mice's weight (grams) and (H) plasma total cholesterol (mg/dL) after 16 weeks of WTD feeding for *Adamts7*^{ECCTL} *Ldlr*^{KO} and *Adamts7*^{ECTG} *Ldlr*^{KO} mice. *n* = 3 - 4 female mice. Statistics were analyzed using a 2-tailed Student's t-test. ****P<0.0001, ***P<0.001, **P<0.01, *P<0.05

SUPPLEMENTAL FIGURE 3

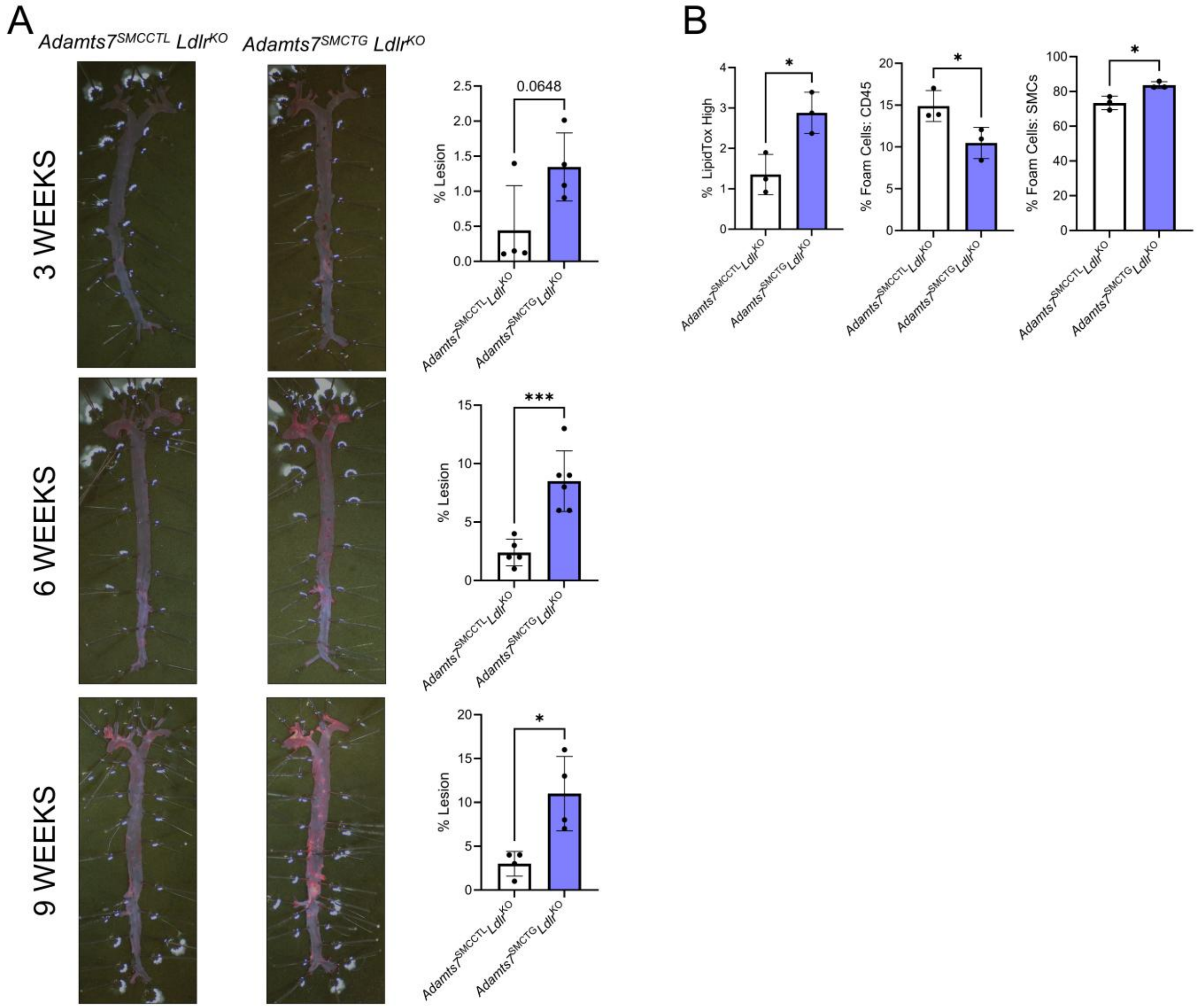

Supplemental Figure 3. Increased atherosclerosis with short durations of WTD feeding in *Adamts7*$^{\mathbf{SMCTG}}$ mice.

(A) Representative images of ORO stained en-face aortas after three, six, and nine weeks of WTD feeding. *n* = 4 - 6 male mice (B) Foam cell analysis of aortas after 3 weeks of WTD feeding. Aortic single-cell suspensions were stained for leukocyte marker CD45 and smooth muscle cell marker CD200 *n* = 3 male mice. **P*<0.05; ****P*<0.001 Statistics were analyzed using a 2-tailed Student's t-test.

SUPPLEMENTAL FIGURE 4

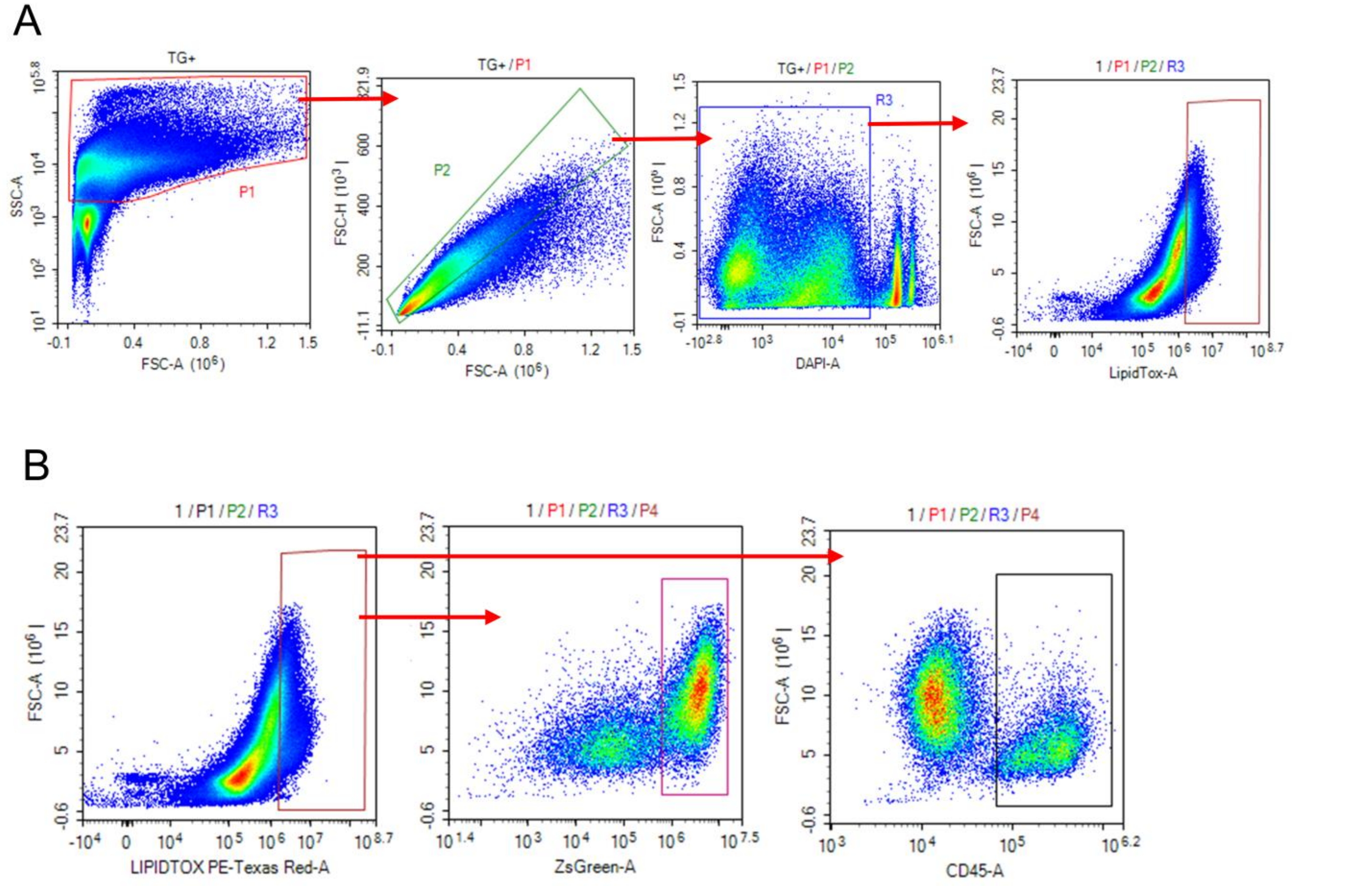

Supplemental Figure 4. Representative gating strategy for foam cells.

(A) Representative flow cytometry gating strategy. Cells were isolated from debris. Singlets and live cells (DAPI negative) were subsequently analyzed. LipidTOX high gate was established using a normal lipidemic C57BL/6J aorta. (B) LipidTOX high foam cells were further assessed for ZsGreen expression and stained for the leukocyte marker CD45.

SUPPLEMENTAL FIGURE 5

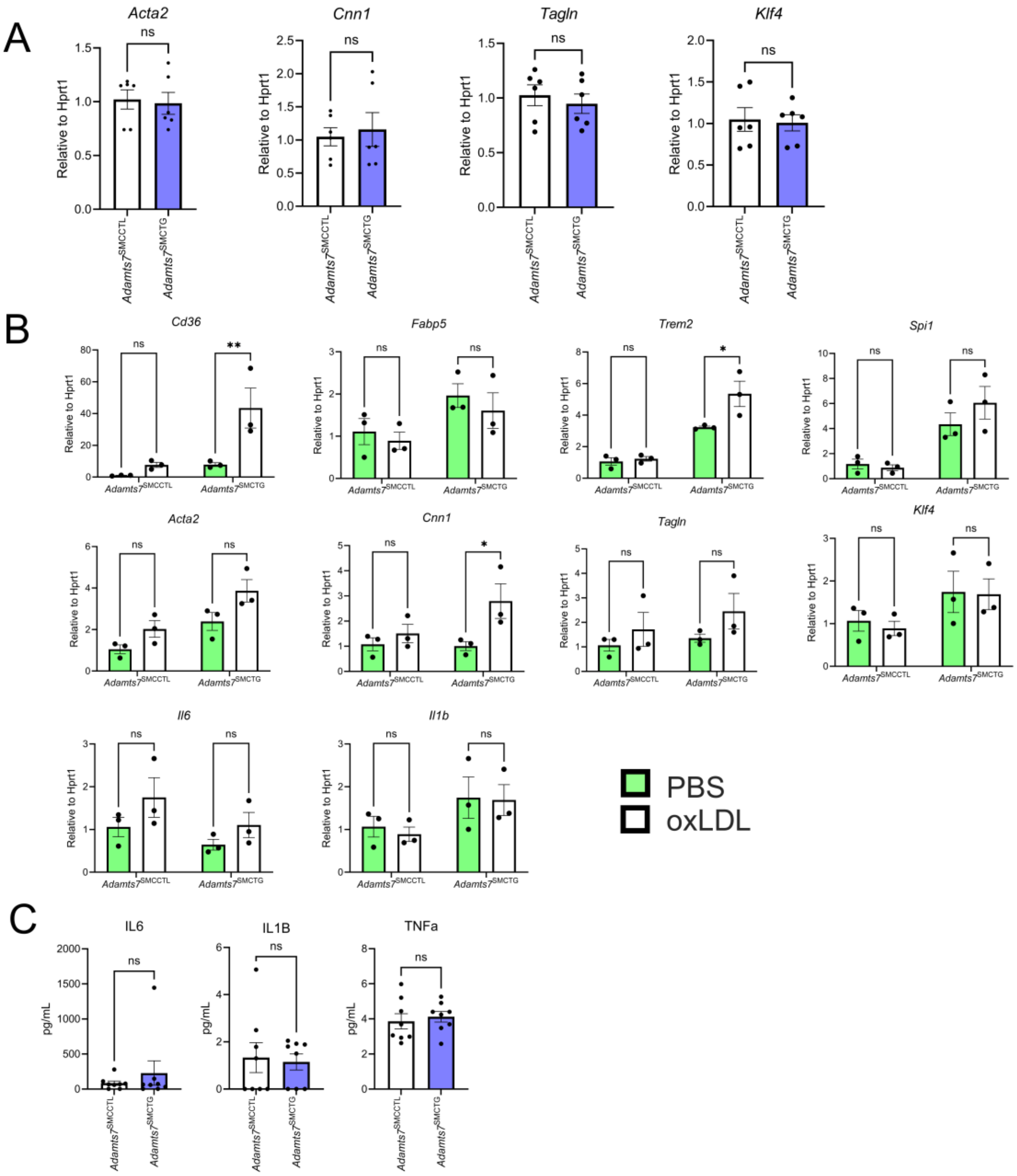

Supplemental Figure 5. Characterization of explanted primary SMCs

(A) qPCR characterization of contractile and modulated genes. *n* = 6. (B) SMCs explanted from *Adamts7*^{SMCCTL} and *Adamts7*^{SMCTG} mice were treated with 100 μg/mL oxLDL for 72 hours, followed by qRT-PCR analysis of the indicated genes. (C) LEGENDplex-based ELISA is used to detect inflammatory cytokines in cell culture-conditioned media. Cells were from *n* = 8 mice. Statistics were analyzed using a 2-tailed Student's t-test or two-way ANOVA with Sidak's post hoc test for multiple comparisons. ****P<0.0001, ***P<0.001, **P<0.01, *P<0.05

SUPPLEMENTAL FIGURE 6

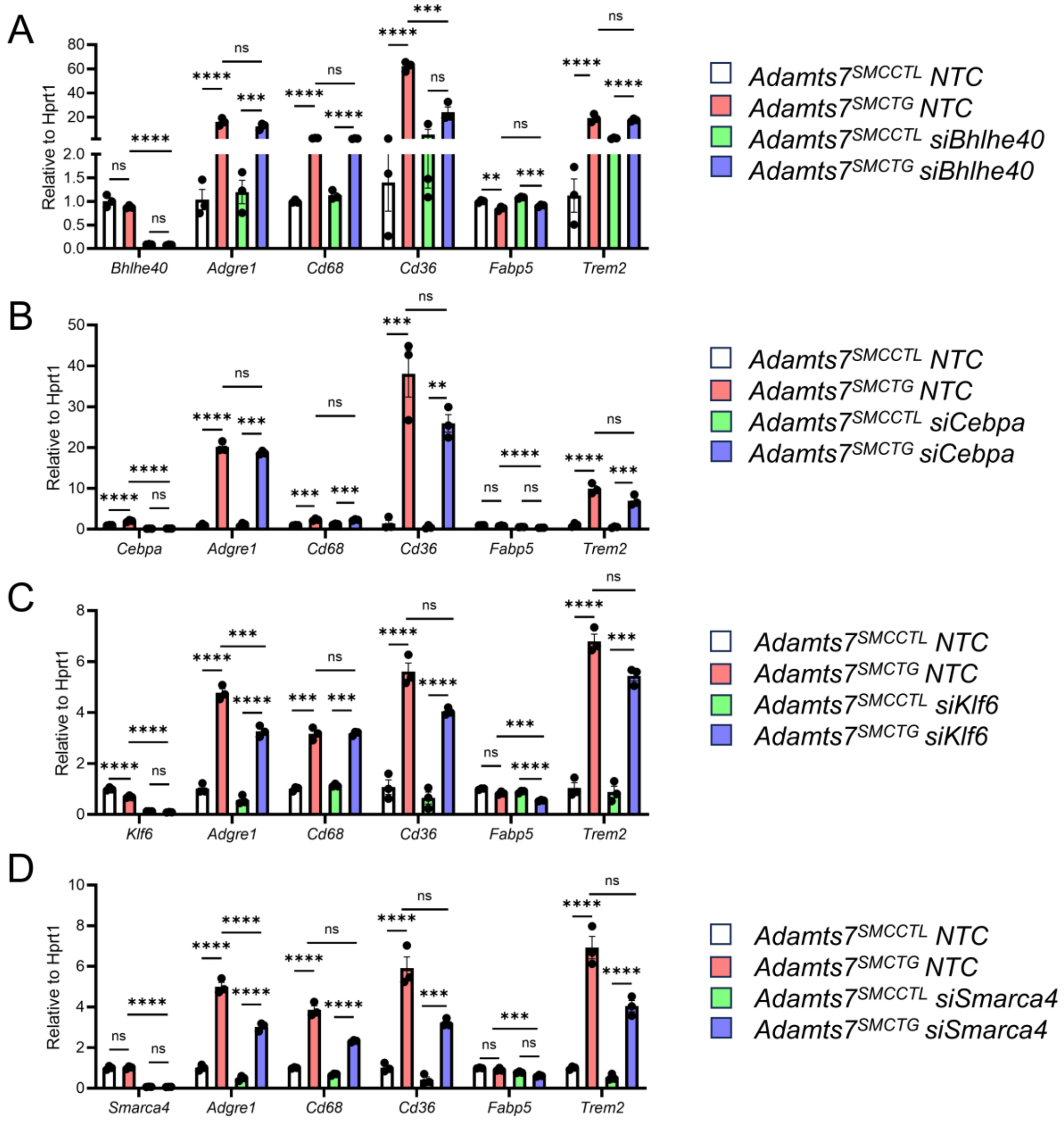

Supplemental Figure 6. Knockdown of candidate transcription factors does not alter *Adamts7* conferred macrophage-like and lipid-handling genes.

Indicated siRNAs (10nM) were used to knock down candidate transcription factors in primary SMCs from *Adamts7*^{SMCCTL} and *Adamts7*^{SMCTG} mice. 48 hours after transfection, cells were harvested for qRT-PCR analysis. ****P<0.0001, ***P<0.001, **P<0.01, *P<0.05 by two-way ANOVA with Bonferroni correction.

SUPPLEMENTAL FIGURE 7

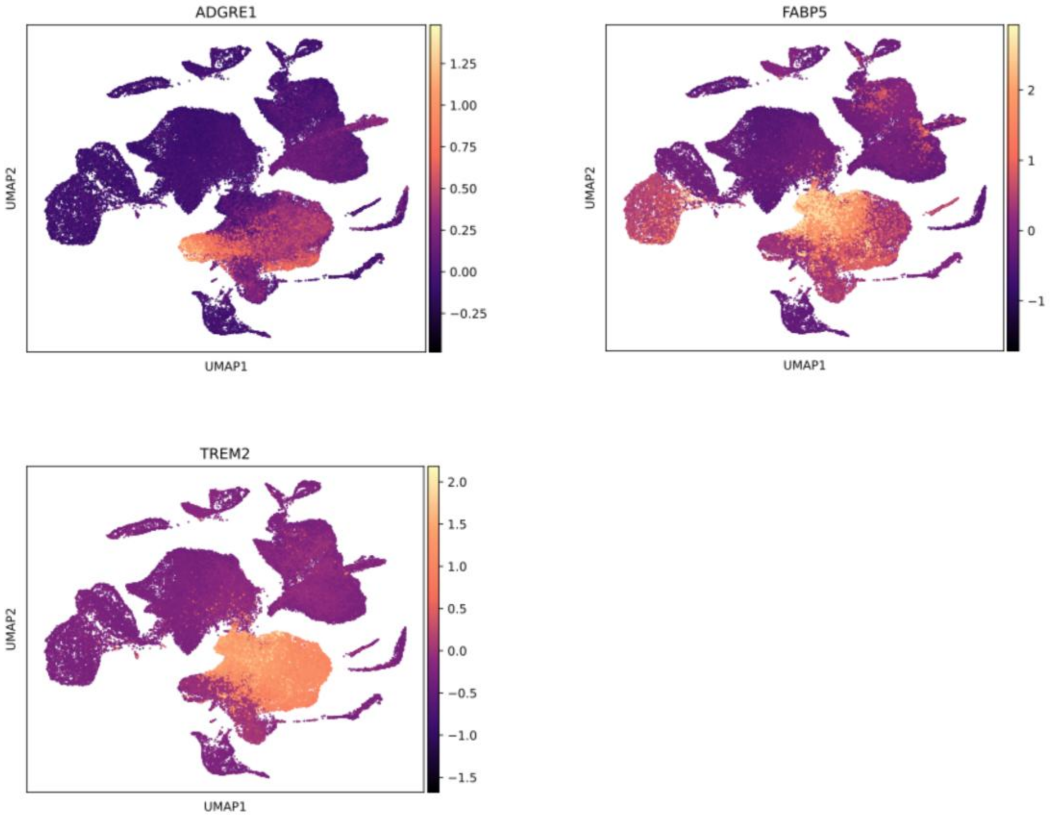

Supplemental Figure 7. Lipid and macrophage-associated gene expression in human carotid atherosclerosis.

Feature plots showing the expression of the indicated genes in scRNA-seq data from human carotid atherosclerotic plaques, as previously published by Bashore AC et al., Arterioscler Thromb Vasc Biol. 2024;44:930–945.

SUPPLEMENTAL FIGURE 8

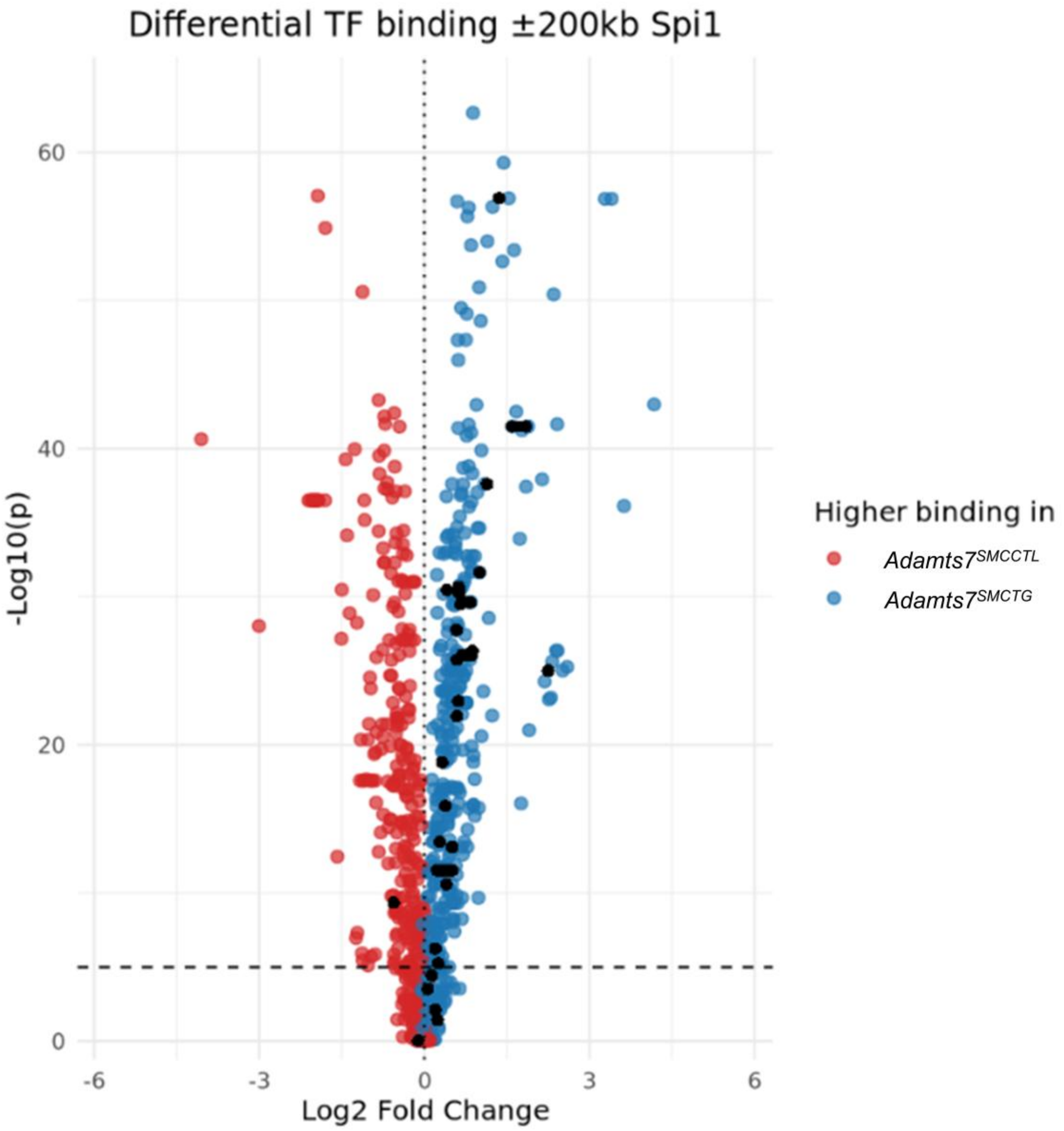

Supplemental Figure 8. ATAC-seq reveals AP-1 binding motifs at the *Spi1* locus.

Volcano plot showing differential chromatin accessibility in *Adamts7*^{SMCCTL} and *Adamts7*^{SMCTG} SMCs within ±200 kb of the *Spi1* transcription start site. Black dots indicate AP-1 binding motifs.

SUPPLEMENTAL FIGURE 9

A

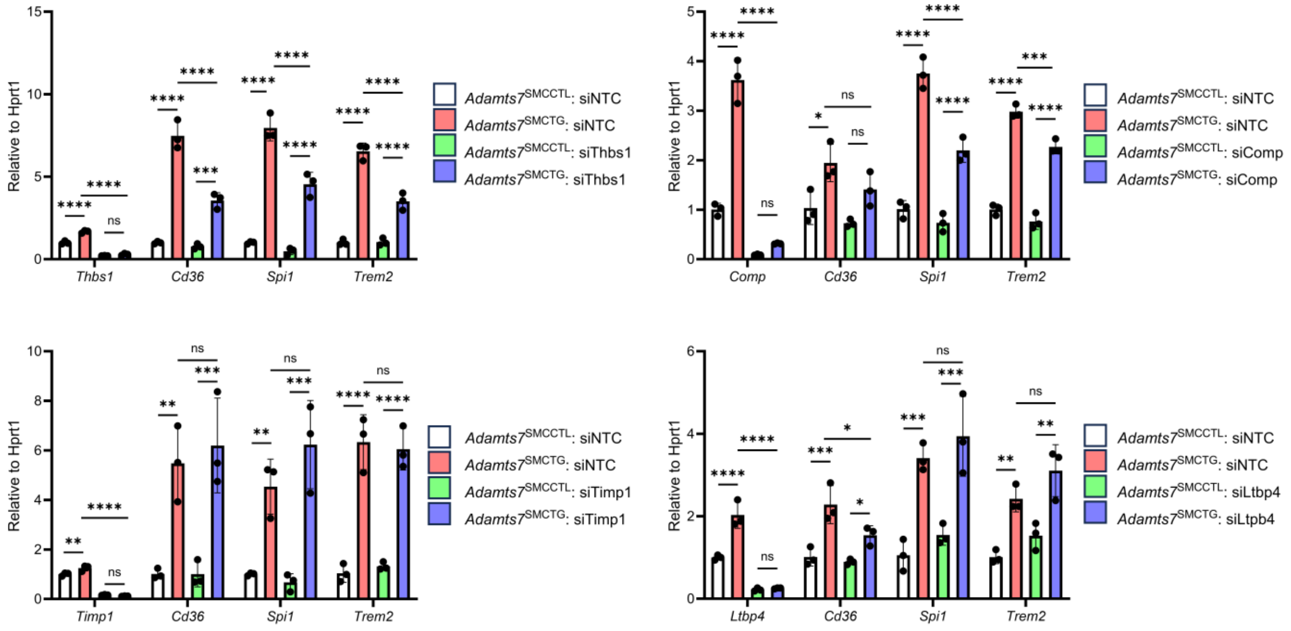

B

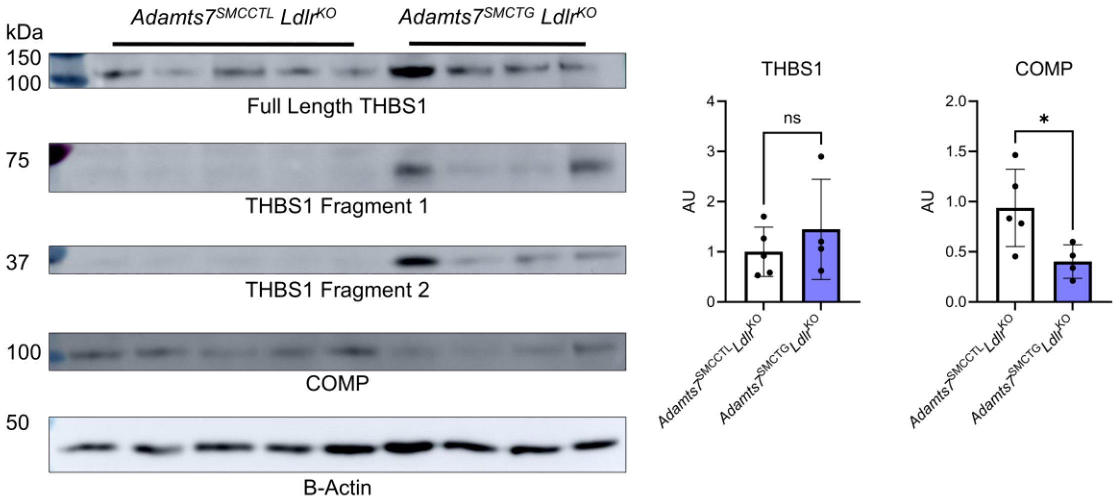

Supplemental Figure 9. Knockdown of candidate ECM substrates to assess their role in SMCs.

(A) Primary SMCs isolated from *Adamts7*^{SMCCTL} and *Adamts7*^{SMCTG} mice were transfected with the indicated siRNAs (10 nM). Cells were harvested 48 hours post-transfection for qRT-PCR analysis. Statistical significance was determined by two-way ANOVA with Sidak's multiple comparisons test: ****P<0.0001, ***P<0.001, **P<0.01, *P<0.05. (B) Western blot of whole-aorta lysates from mice fed a WTD, probed for ECM components THBS1 and COMP (n = 4 - 5 male mice). Quantification of COMP and full-length THBS1 relative to B-actin. Statistical significance was assessed using a two-tailed Student's *t*-test. *P<0.05.
