# Supplementary figures and images for: ADAMTS7 promotes smooth muscle foam cell expansion in atherosclerosis
Source: J Clin Invest. 2026 Jan 29;136(6):e187451. doi: 10.1172/JCI187451 (PMC12987627; doi:10.1172/JCI187451)

## Supplemental Figure 2B

ADAMTS7

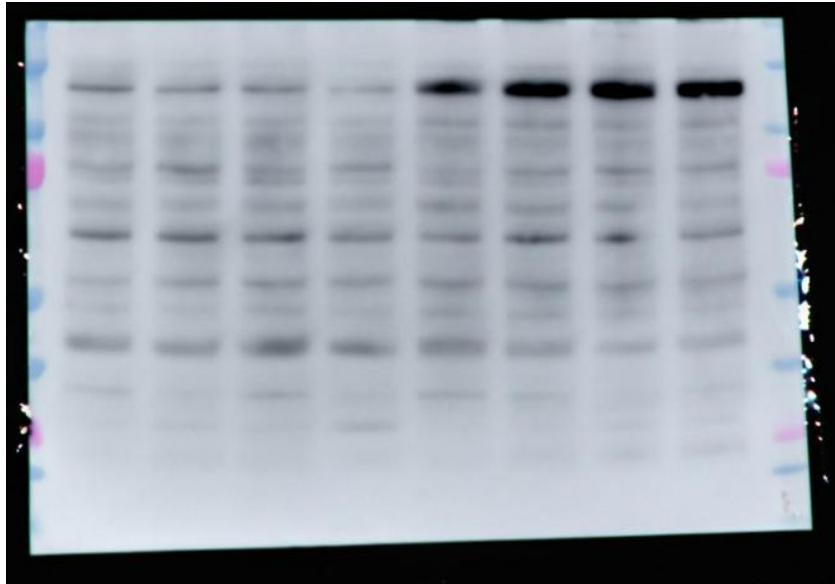

GAPDH

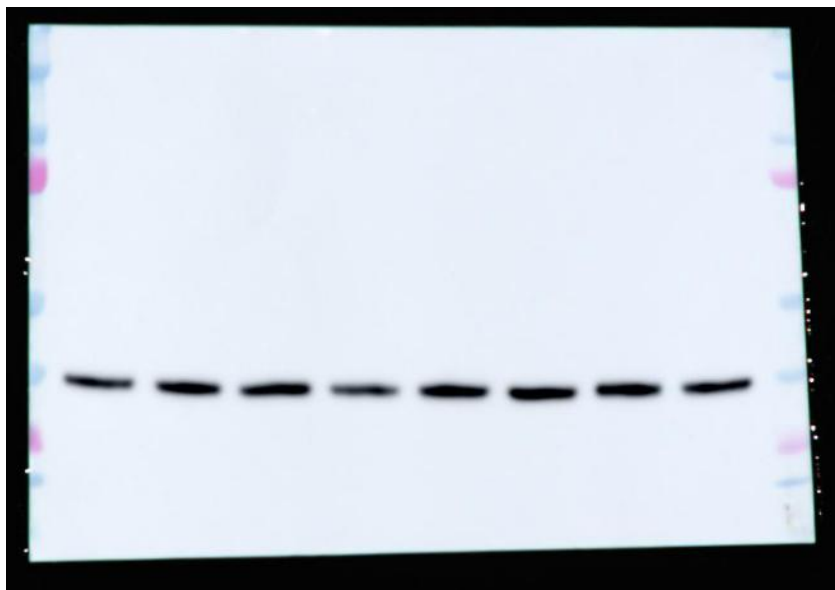

Supplemental Figure 9B

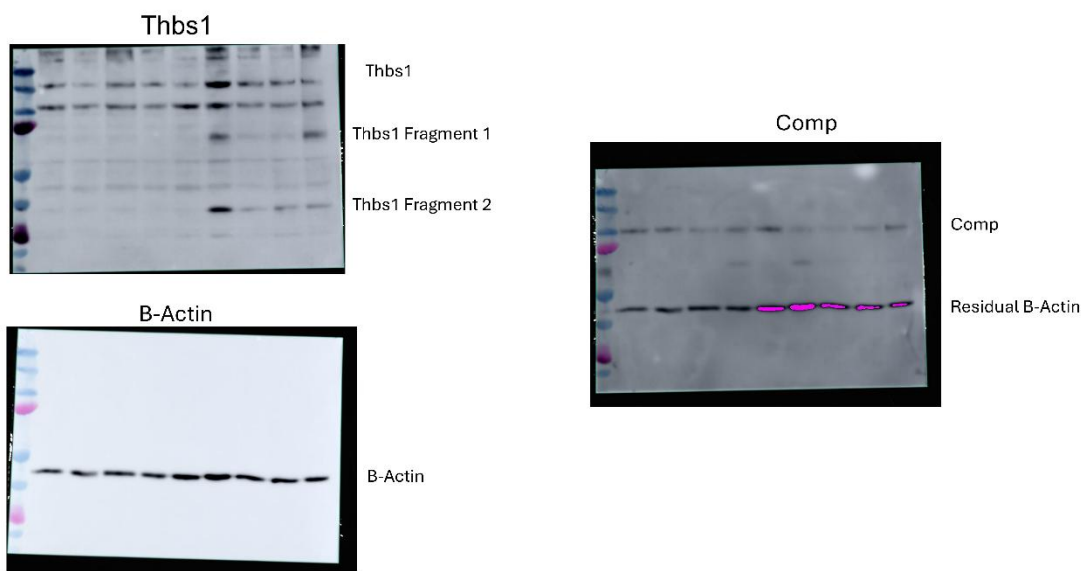

Supplement: Unedited blot and gel images [file jci-136-187451-s209.pdf]
